# Application of Nano-SiO_2_ Reinforced Urea-Formaldehyde Resin and Molecular Dynamics Simulation Study

**DOI:** 10.3390/ma15248716

**Published:** 2022-12-07

**Authors:** Jun Xiao, Dingmeng Guo, Changlei Xia, Taohong Li, Hailan Lian

**Affiliations:** 1 College of Materials Science and Engineering, Nanjing Forestry University, Nanjing 210037, China; 2 Collaborative Innovation Center for Efficient Processing and Utilization of Forestry Resources, Nanjing Forestry University, Nanjing 210037, China; 3 Key Laboratory for Forest Resources Conservation and Utilization in the Southwest Mountains of China, Southwest Forestry University, Kunming 650224, China; 4 Jiangsu Engineering Research Center of Fast-Growing Trees and Agri-Fiber Materials, Nanjing 210037, China

**Keywords:** urea formaldehyde resin, nano-SiO_2_, modification, molecular dynamics simulation

## Abstract

Nano-SiO_2_ is a typical modifier used for urea-formaldehyde (UF) resins to balance the reduced formaldehyde content and maintain bond strength. However, the microstructure of UF resin and the interaction between UF resin and nano-SiO_2_ are microscopic phenomena; it is difficult to observe and study its intrinsic mechanism in traditional experimental tests. In this work, the enhancement mechanism was explored by molecular dynamics simulations combined with an experiment of the effect of nano-SiO_2_ additions on UF resin. The results showed that the best performance enhancement of UF resin was achieved when the addition of nano-SiO_2_ was 3 wt%. The effects caused by different additions of nano-SiO_2_ were compared and analyzed by molecular dynamics simulations in terms of free volume fraction, the radius of gyration, and mechanical properties, and the results were in agreement with the experimental values. Meanwhile, the changes in hydrogen bonding and radial distribution functions in these systems were counted to explore the interaction between nano-SiO_2_ and UF resin. The properties of the UF resin were enhanced mainly through the large number of different forms of hydrogen bonds with nano-SiO_2_, with the strongest hydrogen bond occurring between H_(SiO2)_… O = _(PHMU)_.

## 1. Introduction

Urea-formaldehyde (UF) resin is a polymeric condensation product of the reaction of formaldehyde with urea. Although it has formaldehyde emissions and a lack of resistance to boiling water, it is still the most widely used adhesive in the wood industry due to its cheap raw materials, easy preparation, excellent gluing properties, and good moisture resistance. UF resin adhesives currently account for 90% of the glue used in wood-based panels and 60% of the total consumption of adhesives in the wood processing industry [1]. For environmental reasons, low formaldehyde/urea molar ratio formulation and batch feeding with urea can obtain a UF resin with relatively low free formaldehyde content, but this hurts the bond strength of the resin [2,3,4], and it is difficult to achieve a greater breakthrough. Therefore, researchers have been working to enhance the bonding strength of UF resins and reduce the free formaldehyde content by adding various formaldehyde scavengers.

Nanomaterials are often used for compounding with other materials to improve their physicochemical properties because of their nanoscale scale and extremely high specific surface area, which is a class of urea-formaldehyde resin modifiers with excellent performance [5]. There is a wide range of nanomaterials, such as mineral nanoparticles: clay [6,7] and sodium montmorillonite [8]; carbon-based nanoparticles: carbon nanotubes [9], graphene oxide [10], and various forms of nanocellulose [4,11,12,13]; and nanoparticle oxides: TiO_2_ [14], SiO_2_ [15] and Al_2_O_3_ [16]. These nanomaterials can favorably affect the mechanical strength, thermodynamic properties, and formaldehyde emission of urea-formaldehyde resins but with different emphases. Among them, nano-SiO_2_ has a large surface area and a large number of silanol (Si-OH) groups on the surface which can form strong physical contact with the urea-formaldehyde resin matrix, and the resin strength is greatly improved [1]. Moreover, these surface functional groups can effectively adsorb free formaldehyde inside the resin and are a commonly used modifier for urea-formaldehyde resins [17,18]. While E. Roumeli et al. [15]. synthesized and characterized UF resins with different nano-SiO_2_ additions and showed that nanoparticles’ aggregation in resin increases with increasing nano-SiO_2_ content, excessive aggregation is detrimental to the numerical properties and sheet performance. Therefore, it is necessary to regulate the amount of nano-SiO_2_ addition to obtain the best modification effect.

Moreover, because the microstructure of UF and the interaction between UF and nanoparticles are microscopic phenomena, it is difficult to observe and study its intrinsic mechanism in traditional experimental tests. With the development of computer technologies, simulation tools based on quantum chemical methods [19] and molecular mechanics methods [20,21] have been updated. Microscale information obtained by the computational prediction of molecular models has greatly filled the gap of traditional experimental data and deepened enhancement mechanisms.

Currently, computer simulation techniques have been applied to solve problems in various fields, such as biomolecules [22,23,24], drug development [25,26], energy storage materials [27], adsorbent materials [28,29], and polymers [30,31]. As one of the commonly used computer simulation methods, molecular dynamics (MD) simulations have been widely used to study the mechanical properties of materials, and many researchers have demonstrated the feasibility of this approach [32,33,34].

In this study, the effects of different nano-SiO_2_ additions on the properties of UF resin and plywood were investigated experimentally. Then, the molecular systems of UF resin/nano-SiO_2_ were also constructed for molecular dynamics simulation [35,36] to explain the interaction mechanism between nano-SiO_2_ and UF resin at the molecular level. The purpose of this study is to combine macroscopic and microscopic scales to elaborate the enhancement mechanism of nano-SiO_2_ on UF resin and to refine the enhancement theory to provide guidance for the development of new UF resin with excellent properties.

## 2. Materials and Methods

### 2.1. Materials

Formaldehyde (37%), urea (98%), sodium hydroxide, formic acid, and ammonium chloride used in this experiment were all analytical pure grade and purchased from Shanghai Maclin Biochemical Technology Co., Ltd. (Shanghai, China). Nano-SiO_2_ was purchased from Guangzhou Hongwu Material Technology Co., Ltd. (Guangzhou, China). All reagents were used directly without further purification. The veneer used was *pine wood* spin-cut veneer, width 400 mm × 400 mm, thickness 1.5 mm, made by the laboratory.

### 2.2. Preparation and Performance Test of UF Resin

The UF resin was synthesized by the “alkali-acid-alkali” process with three additions of urea, and the final molar ratio of formaldehyde to urea was 1.5. The nano-SiO_2_ modified UF resin (UF-SiO_2_-1%, UF-SiO_2_-2%, UF-SiO_2_-3%, UF-SiO_2_-4%) was prepared by adding 1, 2, 3, and 4 wt% of nano-SiO_2_ to the resin, respectively. The nano-SiO_2_ and UF resin were stirred at 600 r/min for 10 min to make a homogeneous mixture. The properties of the UF resins were measured according to the Chinese national standard GB/T 14074-2017 “Test method for adhesives and their resins for the wood industry”.

### 2.3. Preparation and Performance Measurement of Plywood

UF resin was added with 5% flour as filler, and 1% ammonium chloride was used as a curing agent. The three layers of pine veneer were staggered according to the grain, and after 30 min of open assembly, they were hot pressed at 110 °C and 1.5 MPa for 4 min. The properties of the prepared plywood were measured according to the national standard GB/T 17657-2013 “Test method for physical and chemical properties of man-made boards and veneered man-made boards”.

### 2.4. Simulation of Configuration Construction

**Figure 1:**
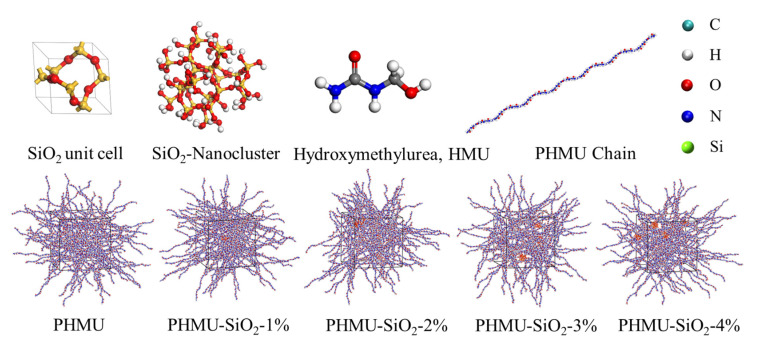
The configurations involved in the study.

All calculations in this study were performed based on Materials Studio 2016 software. The structure of SiO_2_ nanoclusters with a radius of 6 Å was first constructed, and then H atoms were added to the surface O atoms, and −OH was attached to the Si atoms to eliminate the unsaturated bonds on the surface of the nanoclusters. A total of 44 hydroxyl groups existed on the surface of one nano-SiO_2_ cluster. Polymer chains (PHMU) formed by condensation of mono-hydroxymethylurea(HMU) monomer were chosen to simulate UF resin with a degree of polymerization of 40. The effect of different additions was investigated by varying the number of nanoclusters inserted in the simulation box. The pure PHMU simulation box was added with 66 PHMU chains, as in Figure 1. Next, 1, 2, 3, and 4 additional nano-SiO_2_ clusters were added to each simulation box for the nano-SiO_2_-doped system to obtain systems with different mass ratios of SiO_2_ doping (denoted as PHMU-SiO_2_-1%, PHMU-SiO_2_-2%, PHMU-SiO_2_-3%, and PHMU-SiO_2_-4%). The information on the composition of the different simulated systems is shown in Table 1.

### 2.5. Simulation Method

The COMPASS force field [37], which is suitable for studying the structure, mechanics, and thermodynamics of condensed matter systems, was chosen as the simulated force field for this paper. The COMPASS force field divides the total potential energy term into two categories: One is the energy dominated by short-range covalent bonds, which is subdivided into bond stretching energy, bond bending energy, dihedral angle torsion energy, out-of-plane vibration energy, and interaction energy between crossed terms [38]. The other type is the energy dominated by noncovalent interactions, such as Coulomb electrostatic interaction energy and van der Waals interaction energy.

The geometry optimization and equilibrium of the molecular structure and the simulation box were carried out with the computational quality of “fine”, the algorithm chosen for the geometry optimization was the smart method [39], and the calculation of the electrostatic and van der Waals interactions was done by the atom method. Subsequently, annealing simulations in the range from 200 to 350 °C were performed in NVT synthesis to obtain the lowest energy configuration. Then, a dynamic equilibrium of 100 ps at 298 k was performed in the NVT system to converge the energy and temperature of the structure where the time step was set to 1 fs, and the temperature control method was the nose method [40]. After that, the energy and density of the system were further regulated by NPT synthesis, and the pressure was controlled by a Berendsen regulator [41] at 0.1 MPa for 200 ps of kinetic simulation. In the dynamic equilibrium process, the van der Waals force was calculated by the atom method, and the electrostatic force was calculated by the Ewald method.

## 3. Results

### 3.1. Effect of Nano-SiO_2_ Addition on the Properties of UF Resin

**Figure 2:**
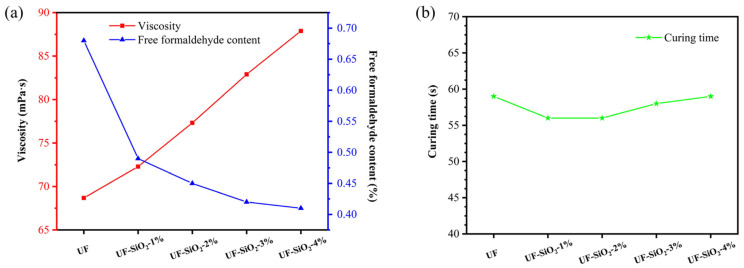
Viscosity and free formaldehyde content of UF resin (**a**); curing time of UF resin (**b**).

The properties of UF resin modified by nano-SiO_2_ would show a series of changes, so they needed to be analyzed and regulated to achieve the desired effect. First of all, the viscosity of UF resin was an important indicator in the applicability of the product. If the viscosity was too low, this would lead to the glue layer easily penetrating the surface of the glued substrate, seriously affecting the bonding strength and bonding effect. The viscosity of UF resin without nano-SiO_2_ was 68.7 mPa·s, and the viscosity was improved after mixing the UF resin with nano-SiO_2_. Meanwhile, the viscosity of the UF resin gradually increased with the addition of nano-SiO_2_ in a certain range (Figure 2a). This may be due to the presence of a large number of unsaturated residual bonds on the surface of nano-SiO_2_ as well as hydroxyl groups in different bonding states, which would form a variety of noncovalent interactions, such as hydrogen bonds and van der Waals forces, with the functional group of the UF resin. In addition, the increase in force between the molecular chain segments and the increase in the resistance to their relative motion was reflected in the increase in the viscosity of the UF resin.

The curing time of UF resin affects the later plywood preparation process and was therefore an important performance indicator. However, from the results of Figure 2b, the effect of the addition of nano-SiO_2_ on the curing time was minimal. This was because the addition of a small amount of nano-SiO_2_ did not change the conditions such as acidity and alkalinity of the system, and the effect of nano-SiO_2_ on the reaction sites of the prepolymer was almost negligible from the microscopic scale, so there was no significant change in the curing time of the UF resin.

Due to a large number of unstable hydroxymethyl and diethylenes ether bonds in the structure of UF resin, it was easy to break and release free formaldehyde to the environment. The high specific surface area of nano-SiO_2_ and the presence of a large number of hydroxyl groups on the surface can effectively adsorb free formaldehyde. As shown in Figure 2a, the free formaldehyde content gradually decreased with an increase in nano-SiO_2_ addition, but the decrease was gradually reduced. The small particle size of nanoparticles was easy to agglomerate, thus affecting the adsorption of free formaldehyde, and there was no great benefit to blindly increasing the addition amount.

### 3.2. Effect of Nano-SiO_2_ Addition on the Performance of UF Resin Plywood

**Figure 3:**
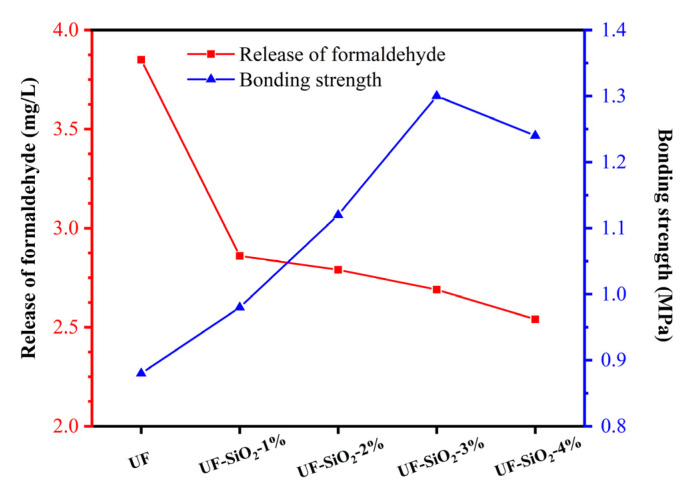
Release of formaldehyde and bonding strength of the panels.

According to GB/T 17657-2013 “Test method for physical and chemical properties of man-made boards and veneered man-made boards”, the formaldehyde emission of the boards prepared with different UF resins was measured by the desiccator method. The formaldehyde emission decreased rapidly with the addition of nano-SiO_2_ (Figure 3). However, with a further increase in nano-SiO_2_ addition, the formaldehyde emission reduction was not satisfactory enough, which was similar to the free formaldehyde content of UF resin. This hindrance may be broken by increasing the homogeneity of the nano-SiO_2_ mixture with the UF resin or by reducing the nano-SiO_2_ particle size.

The shear bonding strengths of the panels are shown in Figure 3, and the bonding strengths of the panels prepared with UF, UF-SiO_2_-1%, UF-SiO_2_-2%, UF-SiO_2_-3%, and UF-SiO_2_-4% were 0.88, 0.98, 1.12, 1.30, and 1.24 MPa, respectively. The addition of nano-SiO_2_ could effectively improve the mechanical properties as well as the bonding ability of UF resins, resulting in a significant increase in the bonding strength of the sheets; this coincided with the results of other researchers [42]. At the 4 wt% addition, the bonding strength decreased compared to the 3 wt% addition, which may also be attributed to the aggregation effect of the nanoparticles. The addition of nano-SiO_2_ exerted beneficial effects on the viscosity of UF resin, the bonding strength, and the release of formaldehyde in plywood.

### 3.3. Molecular Dynamics Simulation

**Figure 4:**
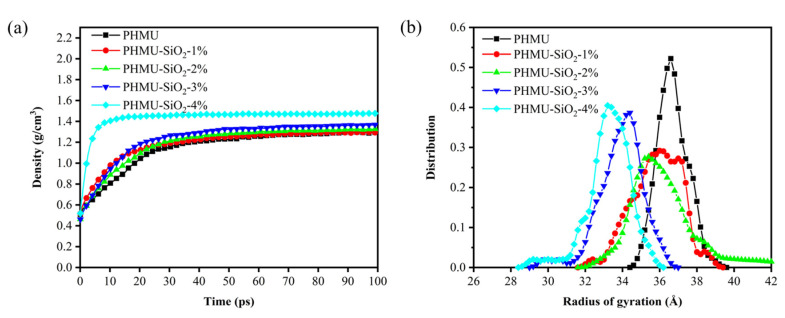
Density (**a**) and radius of gyration (**b**) for different systems after equilibration.

To understand the mechanism of the nano-SiO_2_-enhanced UF resin properties from a microscopic perspective, the trajectories were calculated after the equilibrium of each system to investigate the effect of the addition of nano-SiO_2_ on the properties of the system. Firstly, the density changes in the five systems during the equilibrium to stabilization process was calculated. The equilibrium density of the pure PHMU system was 1.29 g/cm^3^, which was close to the experimental value [43]. This indicates that the COMPASS force field was still reasonable for the description of the system and also shows the credibility of the simulation results. Moreover, the addition of nano-SiO_2_ would fill the network voids of PHMU chains and increase the density of the system (Figure 4a). The radius of gyration reflected the collapse or extension of polymer molecules [44]. The distribution of the radius of gyration of PHMU chains in the pure PHMU system was concentrated around 36.7 Å, as shown in Figure 4b, which indicated that the extension activity of its chain segments was good. With the addition of nano-SiO_2_, the radius of gyration distribution of PHMU chains was gradually smaller, which indicated that the interaction of nano-SiO_2_ with PHMU chains continuously collapses them and effectively limited their motion. Macroscopically, the density and viscosity of the UF resin increased with the addition of nano-SiO_2_.

**Figure 5:**
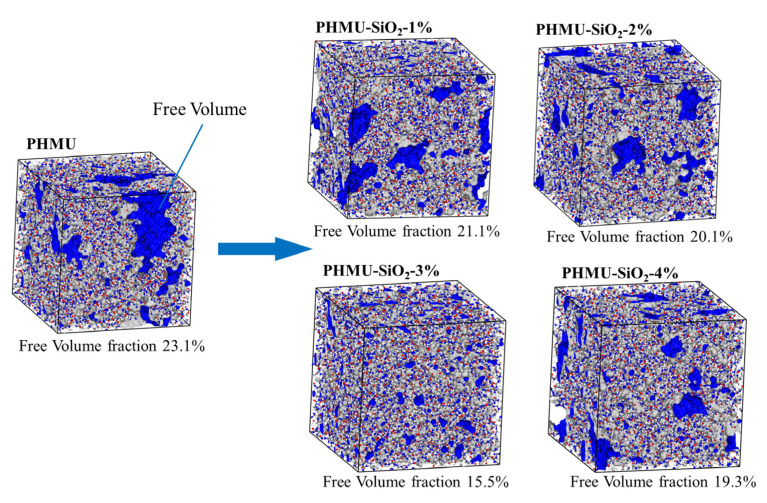
Free volume of the system after equilibrium.

The increasing tendency of density and viscosity were reflected by the variation of free volume for the different systems as well, as seen in Figure 5. The total volume V_sp_ of the simulation box can be divided into occupied volume V_vdW_ (volume occupied by molecular van der Waals surface) and free volume V_f_ (unoccupied volume), and the free volume fraction was the ratio of free volume to total volume. With the addition of nano-SiO_2_, the free volume fraction decreased compared to the pure PHMU system; the system became denser, and the density and viscosity of the material increased. Meanwhile, the lower free volume fraction of the PHMU-SiO_2_-3% system compared to the PHMU-SiO_2_-4% system was due to the better dispersion of the silica nanoparticles in the system.

The mechanical properties of the equilibrated system were calculated using the constant strain energy minimization method, which was used to periodically apply a constant strain of 0.3 wt% to the simulation box, rearrange the atoms and minimize the potential energy of the system, and finally calculate the stiffness matrix of the system *C*_ij_. Since the data of the stiffness matrix of these systems were symmetrically distributed along the diagonal and the diagonal components were much higher than the nondiagonal components, they could be considered isotropic material, so the shear modulus of elasticity *G* was equal to the Lamé constant *μ*. Some mechanical property parameters of the material can be calculated by the following equation:

(1) $$\lambda =\frac{1}{3}\left(C_{11}+C_{22}+C_{33}\right)-\frac{2}{3}\left(C_{44}+C_{55}+C_{66}\right),$$

(2) $$\mu =\frac{1}{3}\left(C_{44}+C_{55}+C_{66}\right),$$

(3) G=μ,

(4) $$E=\mu \frac{3\lambda +2\mu }{\lambda +\mu },$$

The Young’s modulus of the material denoted as *E* can be obtained from Equation (4). Young’s modulus was proportional to the stiffness of the material, and a larger *E* indicates that the material was more resistant to deformation. The mechanical property parameter data of the five systems are summarized in Table 2. The comparison reveals that the addition of nano-SiO_2_ significantly increased the shear elastic model and Young’s modulus of the PHMU system, and the shear elastic modulus of the PHMU-SiO_2_-3% system increased up to 17.4% compared to the pure PHMU system. The mechanical properties of the material started to decrease when the addition amount was higher than 3 wt%, which was consistent with the trend of the bonding strength measured in the experimental section. This was because as the SiO_2_ nanoparticle content increased, the nanoparticles were more likely to agglomerate and thus interact with each other, reducing the interaction with the resin chains and causing a decrease in the local mechanical properties.

To study the form of action inside the system, the hydrogen bonding inside the system during the kinetic process was counted by the H-bonds script listed in Table 3. The cut-off distance between the H donor and the acceptor was set to 2.5 Å, and the cut-off angle of X−H…Y was greater than 150°. The average total number of hydrogen bonds for the five systems PHMU, PHMU-SiO_2_-1%, PHMU-SiO_2_-2%, PHMU-SiO_2_-3%, and PHMU-SiO_2_-4% had an average total hydrogen bonding number of 1888, 1904, 1933, 1966, and 1953, respectively. The average hydrogen bond lengths in each system were not significantly different while the number of hydrogen bonds in the SiO_2_-doped systems was higher than that of the pure PHMU system. This was because the hydroxyl groups on the surface of the nano-SiO_2_ formed a large number of hydrogen bonding interactions with the PHMU chains, which was the main driving force for the improvement of the mechanical properties of the UF resin.

**Figure 6:**
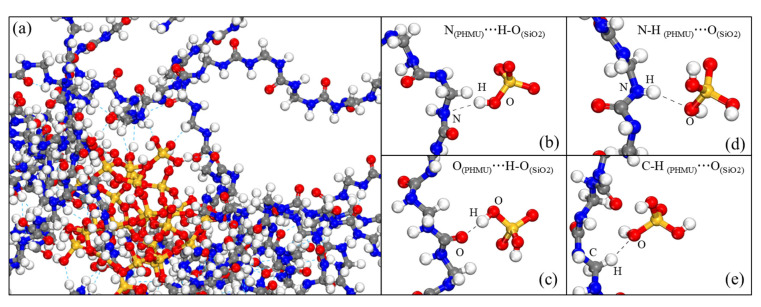
Snapshots from the PHMU-SiO_2_-3% system during the NPT equilibration at 200 ps duration (**a**), hydrogen bonds formed between N atoms of PHMU chains and OH on nano-SiO_2_ (**b**), hydrogen bonds formed between −C = O of PHMU chains and −OH on nano-SiO_2_ (**c**), hydrogen bonds formed between −NH of PHMU chains and O atoms on nano-SiO_2_ (**d**), hydrogen bonds formed between −CH of PHMU chains and O atoms on nano-SiO_2_ (**e**). Legend: the blue, red, grey, and white spheres represent the N, O, C and H atoms, respectively.

The number of hydrogen bonds formed between the nano-SiO_2_ clusters and PHMU chains was also counted, and a single nano-SiO_2_ cluster in the PHMU-SiO_2_-1% system should form about 26 hydrogen bonds with the PHMU chains. However, each nano-SiO_2_ cluster in the PHMU-SiO_2_-4% system formed only 18 hydrogen bonds with the PHMU chains on average. This was because as the number of nano-SiO_2_ in the system increased, the nano-SiO_2_ were more likely to aggregate with each other and reduce the number of surface-active sites. As suggested by Roumeli [15], despite the crosslinking between nano-SiO_2_ and UF resin, the nano-SiO_2_ agglomeration situation still increased with a higher addition amount. This also led to a decrease in the number of total hydrogen bonds and a decrease in mechanical properties in the PHMU-SiO_2_-4% system. The four main forms of hydrogen bonding between nano-SiO_2_ clusters and PHMU chains were N_(PHMU)_…H−O_(SiO2)_, = O_(PHMU)_…H−O_(SiO2)_, N−H_(PHMU)_…O_(SiO2)_, and C−H_(PHMU)_…O_(SiO2)_, as summarized in Figure 6.

**Figure 7:**
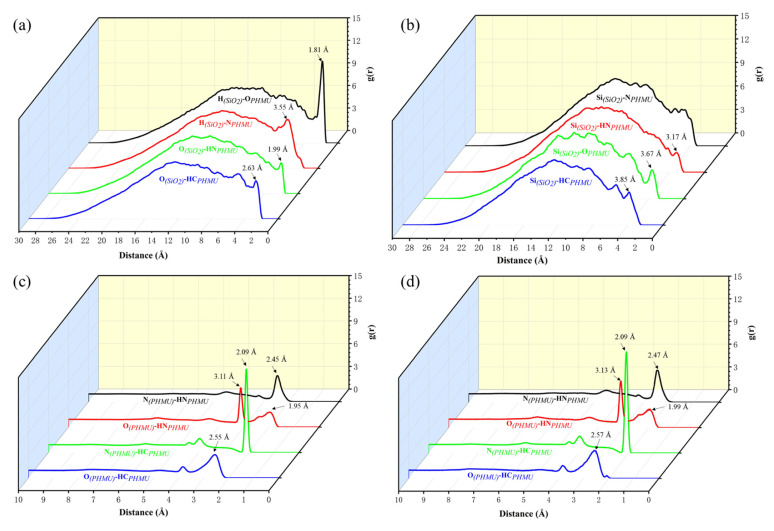
Calculated results of the radial distribution function during the NPT equilibration at 200 ps duration (**a**) H and O atoms of nano-SiO_2_ with PHMU in the PHMU-SiO_2_-3% system; (**b**) Si atoms of nano-SiO_2_ with PHMU in the PHMU-SiO_2_-3% system; (**c**) PHMU and PHMU in the PHMU-SiO_2_-3% system; (**d**) PHMU and PHMU in the pure PHMU system.

To further understand the strength of the interactions within the system, the radial distribution functions (RDF) between the atoms in the system were calculated. A RDF is defined as the probability of the occurrence of another atom at a certain distance from one atom and is often used to analyze the interaction between system components [45]. Figure 7a,b shows the radial distribution functions between H, O, and Si atoms in nano-SiO_2_ and PHMU chains, from which it can be seen that the RDF between H_(SiO2)_…O = _(PHMU)_ has a strong peak at 1.81Å, which was the strongest and most dominant hydrogen bonding interaction between nano-SiO_2_ and PHMU, and the hydrogen bonding between O atoms in nano-SiO_2_ and N−H in PHMU chains was relatively weak with a weak peak at 1.99 Å. Except for these two major hydrogen bonding interactions, the rest of the RDF peaks were at larger distances and generally belonged to the relatively weak van der Waals interactions. Since nano-SiO_2_ and O atoms in carbonyl were prone to strong interactions, the formaldehyde released from the fracture of the UF resin structure was easily adsorbed by nano-SiO_2_, which explains the reduction of free formaldehyde content after nano-SiO_2_ doping.

Figure 7c,d shows the RDF between the atoms of PHMU chains in the nano-SiO_2_ doped system and the pure PHMU system, respectively. The position and intensity of the RDF peaks in the two systems did not change much, which indicated that the addition of nano-SiO_2_ does not destroy the original interaction of PHMU chains. While the main interactions between PHMU chains were hydrogen bonds in the form of C−H…N and N−H…N, the O-atom-dominated interactions were relatively weak.

## 4. Conclusions

The addition of nano-SiO_2_ could significantly improve several performance indexes of UF resins. With an increase in nano-SiO_2_ addition, the viscosity of the UF resin increased continuously, and the free formaldehyde content and formaldehyde emission decreased continuously, but the magnitude was diminished. The bonding strength of the plywood increased with the addition of nano-SiO_2_, reaching a maximum at a nano-SiO_2_ dosage of 3 wt% of the resin and then began to decrease. The curing time of the resin was not significantly affected. Molecular dynamics simulations showed that the addition of nano-SiO_2_ filled the voids between the chain segments, making the system denser and reducing the free volume fraction. At the same time, this reduced the radius of gyration of the PHMU molecular chains and limited their ability to move. A large number of silanol (Si−OH) groups on the surface of nano-SiO_2_ formed many different kinds of hydrogen bonding interactions with PHMU chains, which was the main reason for the improved mechanical properties of the UF resins. RDF analysis showed that nano-SiO_2_ interacts most strongly with the carbonyl O atoms on the PHMU chains, and the free formaldehyde produced was easily adsorbed by this interaction. Excessive addition of nano-SiO_2_ led to an agglomeration effect, which was not conducive to the improvement of UF resin performance, and the optimal addition amount was 3 wt% of the resin mass fraction. However, there are some shortcomings in this paper on the study of the mechanism of nano-SiO_2_-enhanced UF resin. For example, the effects of the different model compounds on the results can be compared, and the interaction of nano-SiO_2_ with UF resin can be analyzed using quantum chemical calculations, which could be further improved in a subsequent study.

## Figures and Tables

**Table 1:** Composition of different simulation systems.

System	Number of PHMU Chains	Number of Nano-SiO_2_ Clusters	Total Number of Atoms in the System
PHMU	66	-	23,958
PHMU-SiO_2_-1%	66	1	24,102
PHMU-SiO_2_-2%	66	2	24,246
PHMU-SiO_2_-3%	66	3	24,390
PHMU-SiO_2_-4%	66	4	24,534

**Table 2:** Summary of mechanical performance parameters of each system.

System	*C*_11_	*C*_22_	*C*_33_	*C*_44_	*C*_55_	*C*_66_	*λ*/ GPa	*μ*/ GPa	*E*/ GPa
PHMU	10.0935	10.2791	8.7662	2.8329	2.6627	2.7714	4.2015	2.7557	7.17
PHMU-SiO_2_-1%	11.3011	8.9072	9.6571	2.8828	2.9239	2.7462	4.2532	2.8510	7.41
PHMU-SiO_2_-2%	10.8068	10.1945	11.3171	2.8998	3.1195	2.8315	4.8723	2.9502	7.74
PHMU-SiO_2_-3%	13.3416	14.2885	14.6164	3.4910	3.0231	3.1938	7.6102	3.2360	8.74
PHMU-SiO_2_-4%	10.2779	10.0658	10.7602	3.0534	2.9722	3.0301	4.3309	3.0185	7.81

**Table 3:** Statistics of hydrogen bonding in different systems calculated from the NPT balance process of 200 ps duration.

System	Total Number of Hbonds	Average Length of Hbonds/(Å)	Hbonds/(SiO_2_-PHMU)
PHMU	1888	2.088	0
PHMU-SiO_2_-1%	1904	2.080	26
PHMU-SiO_2_-2%	1933	2.075	48
PHMU-SiO_2_-3%	1966	2.054	69
PHMU-SiO_2_-4%	1953	2.072	72

## Data Availability

The data that support the findings of this study are available from the corresponding author upon request.
